# Response letter to “Venovenous extracorporeal membrane oxygenation initiation and reduction in vasopressor requirements”

**DOI:** 10.1016/j.aicoj.2026.100102

**Published:** 2026-06-19

**Authors:** Bernhard Nagler, Thomas Staudinger

**Affiliations:** Medical University of Vienna, Department of Medicine I, Intensive Care Unit 13i2, Waehringer Guertel 18-20, 1090 Vienna, Austria

To the Editor,

We read with great interest the correspondence by Labanca et al. [1] regarding our article, “Vasopressor Requirements after Initiation of Venovenous Extracorporeal Membrane Oxygenation in Patients with Severe Respiratory Failure” [2]. We appreciate their valuable insights into the physiological mechanisms of hemodynamic recovery and their agreement with our primary finding that the correction of respiratory acidosis is a key driver of hemodynamic stability. Importantly, the findings of our study are in line with their own research on this topic [3] and a similar study by Odish et al. [4].

The authors correctly point out that the haemodynamic benefits of extracorporeal CO₂ removal may manifest within the first few hours of support. However, the immediate peri-cannulation period is often characterised by procedural interventions such as increased sedation requirements and fluid administration that can obscure the underlying physiological signal. We designed our statistical methodology to account for these confounders as much as feasible given the retrospective nature of the study. Therefore, we consider it crucial to regard a sufficient time span before ECMO initiation, even if this is in many cases limited by the urgency of establishing V-V ECMO support leading to short times between admission to the ECMO centre and cannulation. Analysing only the post-ECMO period may overestimate haemodynamic effects by ignoring high vasopressor needs potentially driven by peri-cannulation sedation. Our study design utilised 24-h aggregate data and data from before ECMO initiation specifically to mitigate this "procedural noise" and to assess the net haemodynamic stabilisation achieved after the initial transition phase.

Regarding the statistical concern of collinearity among pH, PaCO₂, and bicarbonate, we fully share the authors’ caution. To mitigate this, we did not include serum bicarbonate in the model due to its direct mathematical dependence on the other two variables. Furthermore, as illustrated in e-Fig. 1 of our original article's Supplementary material [2], we formally tested for residual multicollinearity using Variance Inflation Factors (VIF). The VIFs for all covariates - including arterial pH and PaCO₂ - remained well below the conservative threshold of 5 (all <3.5), confirming that these variables contributed independent information to the model despite their physiological relationship.

As outlined in our discussion, we agree that our study is limited by the lack of detailed echocardiographic or pulmonary artery catheter measurements. As demonstrated in several previous studies specifically focused on right ventricular (RV) haemodynamics during V-V ECMO [[5], [6], [7], [8]], the concept of “RV unloading” is central to haemodynamic improvement. Our findings should be regarded as observational confirmation that this physiologically expected improvement indeed occurred in the routine clinical setting, when accounting for potential confounders. We emphasise that in our study, the Vasoactive-Inotropic Score served as a surrogate for overall hemodynamic status but was not intended to be a standalone clinical variable for guiding decision-making regarding the indication or configuration of ECMO. We fully agree that such clinical decisions must result from careful haemodynamic phenotyping, including the assessment and monitoring of RV function.

## CRediT authorship contribution statement

BN: original draft. TS: supervision and editing.

## Funding

No third-party funding was received.

## Availability of data and material

The datasets used and/or analysed during the current study are available from the corresponding author on reasonable request.

## Declaration of competing interest

TS: speaker fees: Getinge, Xenios. BN: no conflicts of interest to disclose.

The authors declare the following financial interests/personal relationships which may be considered as potential competing interests:

Thomas Staudinger reports a relationship with Getinge AB that includes: speaking and lecture fees. Thomas Staudinger reports a relationship with Xenios AG that includes: speaking and lecture fees.

If there are other authors, they declare that they have no known competing financial interests or personal relationships that could have appeared to influence the work reported in this paper.
